# Nitroglycerine in HUTT - An explosion in Our Understanding of Unexplained Syncope?

**DOI:** 10.1016/s0972-6292(16)30688-x

**Published:** 2013-11-15

**Authors:** Syamkumar Divakara Menon, Carlos A Morillo

**Affiliations:** Arrhythmia Services, Division of cardiology, McMaster University, Hamilton Health Sciences, Hamiltn, ON

**Keywords:** Nitroglycerine, HUTT, tilt table testing

Undiagnosed syncope is always a perplexing and challenging diagnostic problem that clinician face daily. Vasovagal syncope (VVS) remains the most common cause of syncope especially in the absence of significant structural heart disease and ECG abnormalities. Syncope is a frequent cause of consultation and accounts for about 3% of Emergency room admissions and 1% to 6% hospital admissions in the US [1-4]. Kenny et al introduced head-up tilt table (HUTT) testing in 1986 improving our understanding of VVS and providing a useful diagnostic test with an acceptable diagnostic yield [6] HUTT is useful to determine an individuals susceptibility for VVS, allowing reproduction of the symptoms in a safe and controlled environment [5].The initial HUTT protocols entailed the use of a passive unmedicated orthostatic challenge, however these protocols were time consuming and had a low diagnostic yield in patients presenting with unexplained syncope [7].

Almost two decades ago several investigators shortened the passive tilt phase and associated either isoproterenol, or nitroglycerine, to circumvent this limitation. Isoproterenol was the first agent used and evidence of increased diagnostic yield with a modest loss in specificity particularly at doses above 2.5 mcg/min has been reported and is recommended by the latest ESC guidelines [5]. Low dose isoproterenol when combined with shorter duration of drug free HUTT yielded comparative sensitivity without compromising specificity [8].Occasionally, isoproterenol can be associated with adverse events such as arrhythmias and angina, however these events are rather rare at mean doses under 2 mcg/min. Further studies have recommended the use of low-dose isoproterenol primarily in patients < 50 and nitrates in patients > 50 years of age [9].

For the above reasons isoproterenol is primarily used in younger patients presenting with unexplained syncope and nitrates have been promoted in older subjects.[10] Nitrates were introduced as an alternative to isoproterenol during HUTT by Raviele et al [11] in 1993. Since then several investigators have reported the use of nitrates either in oral, spray or intravenous administration. The precise mechanisms by which nitrates improve the diagnostic yield of HUTT is multifactorial and involve a complex interaction of reduced venous return, baroreceptor and neurohormonal activation [5]. A central action of nitrates also has been postulated [13]. A recent meta-analysis of HUTT in patients with unexplained syncope reported a significant improvement in diagnostic yield with use of nitrates while maintaining specificity compared to high dose isoproterenol [14].

In this issue of the journal, Asati et al report the results of a non-randomized study comparing 2 protocols of nitrate augmented HUTT [15]. Standard protocol included administration of sublingual nitroglycerine after 45 minutes of passive or drug free phase of HUTT, and continuation for another 15 minutes. Protocol B included a 5 minute rest phase after the passive phase HUTT by returning the patient to the supine position, after which nitrates were administered in the supine position, and the patient was tilted for another 15 minutes in both arms.

The main finding of this study was that there were no significant differences between the 2 protocols regarding diagnostic yield, specificity and time to syncope during either the passive or augmented HUTT phases. Similarly, there were no significant differences in specificity but a slight non-significant reduction in diagnostic yield was reported in protocol B during HUTT phase.

This study has a small sample size and could be a limiting factor to accept these findings in prima facie, nonetheless this finding may be hypothesis generating and may support the multifaceted action of nitrates in improving the diagnostic yield and specificity of HUTT. Which is the best methodology to perform a reproducible HUTT protocol? This issue remains largely debated but the ESC 2009 provides an evidenced based recommendation regarding the duration, inclination degree and pharmacological agents that improve diagnostic yield of HUTT [5]. Briefly, no resting phase, i.e returning to the supine position is recommended as this extends unnecessarily HUTT duration and may reset baroreceptor response to orthostatic challenge. Similarly duration of guideline recommended HUTT protocols is between 20 to 30 minutes. If the action of nitrates was merely venodilatory, one would have expected a lower positive response in group 2 compared to group1.

Total HUTT duration should be around 30 minutes with a passive phase lasting between 15-20 based on the fact that the mean time to syncope in most studies is 20 +/- 5 minutes. In the current study duration of HUTT was unnecessarily prolonged as it is well established that longer duration does not increase diagnostic yield and prevents the wider use of HUTT due to limited resources. One of the initial objectives of adding pharmacological challenge during HUTT was to overcome the limitation of prolonged procedure times.

Macedo et al reported yet another modification of nitrate facilitated HUTT in which they compared standard protocol to a shortened protocol with nitrate potentiated phase without a passive phase. Shortened nitrate potentiated protocol was not inferior to standard protocol with regard to sensitivity, specificity and accuracy. Further more a shortened protocol allowed faster diagnosis and was better tolerated [16].

The present study will unlikely change clinical practice, but may provide additional insight to our understanding of the pathophysiology and mechanisms of pharmacological augmentation of HUTT diagnosis of VVS. It is important to stress that the diagnosis of unexplained syncope remains being a clinical one and a negative HUTT does not eliminate the possibility of VVS as the etiology of syncope. Addition of tools such as the Calgary Syncope Symptom Score have been shown to provide excellent diagnostic accuracy and should be routinely used in patients with recurrent explained syncope and little evidence of structural heart disease.

In summary the current study provides newer insights into the benefits of pharmacologically provoked HUTT in patients with unexplained syncope suggesting that nitrates increase diagnostic yield in patients presenting with unexplained syncope.
